# Alleviating Pain in Oculoplastic Procedures by Reducing the Rate of Injection of Local Anaesthetic

**DOI:** 10.2174/1874364101509010156

**Published:** 2015-11-04

**Authors:** Aditi Gupta, Paul J. Tomlins, Aaron T.W. Ng, Tristan T.Q. Reuser

**Affiliations:** 1 Department of Ophthalmology, Birmingham and Midland Eye Centre, West Midlands- B18 7QH, UK; 2 Department of Ophthalmology, Gloucestershire Hospitals NHS Foundation Trust, Sandford Road, Cheltenham, Gloucestershire GL53 7AN, UK; 3 Department of Ophthalmology, Heart of England NHS Trust, Bordesley Green East, Birmingham, West Midlands-B9 5SS, UK

**Keywords:** Local anaesthetic, oculoplastic, pain, rate of injection, visual analog score scale.

## Abstract

**Purpose:**

To investigate whether the rate of infiltration of local anaesthetic influences the pain or efficacy of local anaesthesia in oculoplastic surgery.

**Methods:**

A prospective observational study on consecutive patients undergoing a variety of oculoplastic procedures under local anaesthesia. An observer recorded the rate of injection of local anaesthetic during each procedure. The same mixture of local anaesthetic and the same needle gauge was used in all cases. Patients were asked to rate the pain of both the injection and the surgery using a visual analog scale (VAS).

**Results:**

77 consecutive patients were observed, 39/77 (50.6%) patients were female and the average age was 63.5 years (range 31-94). A statistically significant correlation was found between the rate of injection and the VAS score from the injection (p<0.0001, r=0.42). There was no significant correlation between the injection rate and the VAS score from the procedure itself (p=0.25, r=0.13). Additionally, a significant correlation was found between the injection VAS score and the procedure VAS score (p=0.0002, r=0.42).

**Conclusion:**

The slower the rate of injection of local anaesthetic, the less pain was reported by the patient from the injection itself. Indeed the perception of pain from the surgery overall was significantly related to the pain felt during the injection, highlighting the importance of minimising pain during the injection of the local anaesthetic. We conclude that slowing the rate of injection is an effective way of alleviating pain from administration of the anaesthetic.

## INTRODUCTION

Over recent years, there has been a trend for oculoplastic surgery to be performed under local anaesthetic. Local infiltration of anaesthetic agents can be painful and can negatively impact the patient’s overall experience. Several techniques have been previously described to reduce pain from the infiltration of local anaesthetics. Warming of the local anaesthetic agent prior to injection has been controversial with conflicting evidence [1, 2].Alkalinisation of local anaesthetic solutions with sodium bicarbonate has been shown to be particularly useful where the pain of local anaesthetic injection may not be well tolerated such as in large areas of infiltration [3-5].

The aim of our study was to investigate whether the rate of infiltration of local anaesthetic influenced the pain of the injection or the efficacy of the anaesthesia.

## METHODS

Consecutive patients who were undergoing a variety of oculoplastic procedures were observed. Data from four different surgeons were included, in order to ensure variability in the injection rate. All patients received an equal mix of 2% lidocaine hydrochloride (Hameln Pharmaceuticals Ltd., Gloucester, UK) and 0.75% levobupivacaine hydrochloride (Abbott Laboratories Ltd., Ireland) with adrenaline 1:100,000 (Antigen Pharmaceuticals, Ireland). There were no buffers added to the mixture and the solution was kept at room temperature. The local anaesthetic was injected using a 5 mL syringe *via *a 30-gauge needle.

An observer recorded the time taken in seconds to inject the local anaesthetic and the volume of anaesthetic. The time of injection was taken from the total time the needle was in the skin and the plunger was being depressed. Rate of injection was calculated by dividing the volume of anaesthetic injected by the total injection time.

Postoperatively, each patient was asked to subjectively assess the pain experienced during both the infiltration of the anaesthetic agent and during the surgery itself using two 10-cm visual analog scales (VAS) with 0 cm representing no pain or discomfort and 10 cm representing maximum pain or discomfort.

Statistical analysis was performed using GraphPad Prism version 5.0 b for MacOSX, GraphPad Software, San Diego California USA, www.graphpad.com. Spearman’s Rank Correlation was used to test for significance of continuous variables; Kruskal-Wallace was used to test for difference between groups.

## RESULTS

There were 38 males and 39 females with an age range of 31-94 years (mean= 63.5; SD 15.9). Upper lid, lower lid and medial canthus were the most common sites of injection. The time taken to inject local anaesthetic ranged from 10 seconds to 345 seconds. Injection rate varied from 0.0024 mL/s to 0.104 mL/s with a mean injection rate of 0.031 mL/s (SD 0.027 mL/s). The mean injection VAS score was 1.69 (range 0-7.5, SD 1.90) and the mean procedure VAS score was 0.96 (range 0-10, SD 1.73).

**Fig. (1):**
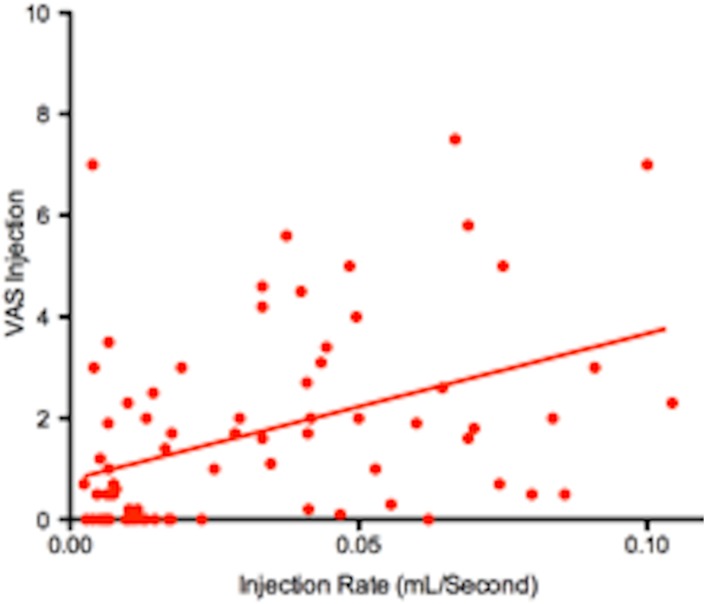
Injection rate versus injection VAS score. Slower the rate of injection, lower the VAS score (p<0.0001).

**Fig. (2):**
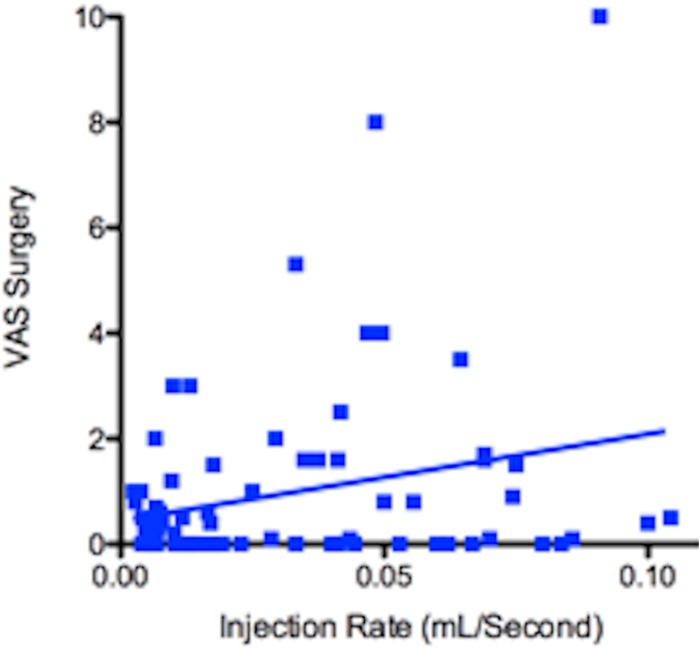
Injection rate versus surgery VAS score. (p=0.2488).

Rate of injection positively correlated with VAS score for injection (Fig. **1**) and the correlation is statistically significant (p<0.0001, r=0.42). Rate of injection positively correlated with VAS score for surgery (Fig. **2**) but the correlation was not statistically significant (p=0.2488, r=0.13).

There was also a significant correlation between the VAS score of the injection and the VAS score of the surgery itself (p=0.0002, r=0.42).

There was no significant correlation between the age of the patient and pain from either the injection (p=0.2011, r=-0.15) or the surgery itself (p=0.5702, r=0.07).

Kruskal-Wallis analysis was performed to investigate whether the pain from the injection was worse if surgery was performed at different sites. The VAS score did not differ significantly at different surgical sites.

## DISCUSSION

In our cohort, we have found that the rate of infiltration of anaesthetic agent is significantly related to the pain of the injection as subjectively reported by the patient.

The choice of agent and mode of infiltration is largely influenced by a surgeon’s experience and assessment of the evidence base. In addition, other constraints such as time and cost play a part. Regardless, alleviating pain and anxiety during the procedure is the prime concern of all surgeons.

A large survey of dental and general surgeons concluded that relatively few surgeons undertook the practice of warming or buffering local anaesthetic solutions. They reasoned that this could be due to a lack of knowledge, or the logistical problems of warming the solutions [6].

It is hypothesised that pain experienced during the infiltration of local anaesthetic may, in part, be due to expansion of tissues. Injecting slowly may therefore have a role in minimising pain. A significant relationship between the injection rate of an anaesthetic agent and the pain experienced from the injection has been demonstrated for penile nerve blocks [7]. Conversely, patients receiving local anaesthetic injection into the dorsum of the hand showed no significant change in injection pain on reducing injection speed [8].

Double- blind randomised controlled trial carried out by Kanna *et al. *[9] investigating the effect of speed of injection on anaesthetic efficacy in inferior alveolar blocks showed complete anaesthesia with slow injections compared to rapid injections. The authors hypothesised a deeper penetration of the local anaesthetic into the nerve trunk as a result of slowing the rate of injection speed [9]. In dental anaesthesia, computer controlled injection devices, such as the Wand™ (CompuDent and Single Tooth Anaesthesia System) allow the surgeon to accurately control the injection rate ensuring a slow and steady injection reducing pain [10] and improving drug delivery and patient comfort [11].

Our data has demonstrated a positive correlation between the rate of injection and pain experienced during anaesthetic infiltration. We believe that the speed of injection is an important factor and further studies are required to investigate the issue. The importance of reducing the pain experienced by the patient during surgery is highlighted by our finding that the patient’s perception of pain from the surgery was significantly affected by their perception of pain from the anaesthetic infiltration.

Pain is a highly subjective phenomenon and a number of factors affect the patient’s reported degree of pain, including age, and the site of surgery. Our study did not show significant statistical correlation between age, site and pain from injection. Other limitations of our study include a small sample size, lack of randomisation and lack of standardisation of incidental preoperative analgesia use.

Nevertheless our study is, to our knowledge, the first to demonstrate a significant reduction of pain from anaesthetic infiltration by lowering the rate of anaesthetic infiltration in oculoplastic surgery. Further randomised controlled trials are needed to study the effect of the rate of injection on the patient’s perception of pain from the infiltration of local anaesthetics.

## CONCLUSION

Our study has shown that perception of pain from the surgery overall was significantly related to the pain felt during the injection, highlighting the importance of minimising pain during the injection of the local anaesthetic. Slowing the rate of the injection is an effective way of alleviating pain from the administration of the local anaesthetic.
